# CALCOCO2/NDP52 and SQSTM1/p62 differentially regulate coxsackievirus B3 propagation

**DOI:** 10.1038/s41418-018-0185-5

**Published:** 2018-08-28

**Authors:** Yasir Mohamud, Junyan Qu, Yuan Chao Xue, Huitao Liu, Haoyu Deng, Honglin Luo

**Affiliations:** 1 Centre for Heart Lung Innovation, St. Paul’s Hospital and Department of Pathology and Laboratory Medicine, University of British Columbia, Vancouver, BC V6Z 1Y6 Canada; 2 Centre for Infectious Disease, West China Hospital, Sichuan University, 610041 Chengdu, Sichuan China; 3 Department of Vascular Surgery, Renji Hospital, Shanghai Jiao Tong University School of Medicine, 200030 Shanghai, China

**Keywords:** Macroautophagy, Microbiology

## Abstract

Cell autonomous immunity is the ability of individual cells to initiate a first line of host defense against invading microbes, such as viruses. Autophagy receptors, a diverse family of multivalent proteins, play a key role in this host response by detecting, sequestering, and eliminating virus in a process termed virophagy. To counteract this, positive-stranded RNA viruses, such as enteroviruses, have evolved strategies to circumvent the host autophagic machinery in an effort to promote viral propagation; however, the underlying mechanisms remain largely unclear. Here we studied the interaction between coxsackievirus B3 (CVB3) and the autophagy receptor SQSTM1 (sequestosome 1)/p62 and CALCOCO2/NDP52 (calcium binding and coiled-coil domain-containing protein 2/nuclear dot 10 protein 52). We demonstrated that SQSTM1 and CALCOCO2 differentially regulate CVB3 infection. We showed that knockdown of SQSTM1 causes increased viral protein production and elevated viral titers, whereas depletion of CALCOCO2 results in a significant inhibition of viral growth. Both receptors appear to have a role in virophagy through direct interaction with the viral capsid protein VP1 that undergoes ubiquitination during infection. Further investigation of the proviral mechanism of CALCOCO2 revealed that CALCOCO2, but not SQSTM1, suppresses the antiviral type I interferon signaling by promoting autophagy-mediated degradation of the mitochondrial antiviral signaling (MAVS) protein. Moreover, we demonstrated that viral proteinase 2A-mediated cleavage of SQSTM1 at glycine 241 impairs its capacity to associate with viral capsid, whereas cleavage of CALCOCO2 by viral proteinase 3C at glutamine 139, generates a stable C-terminal fragment that retains the proviral function of full-length CALCOCO2. Altogether, our study reveals a mechanism by which CVB3 targets selective autophagy receptors to evade host virophagy.

## Introduction

Autophagy is paramount to cellular health and its dysfunction is implicated in the pathogenesis of many human diseases. Although autophagy is widely considered a cellular strategy to remove damaged proteins/organelles and to eliminate invading pathogens, its reciprocal regulation by the latter has only recently come to light. In particular, non-enveloped, positive-stranded RNA viruses, such as enteroviruses, have emerged as hijackers of the autophagy pathway, to ultimately usurp its abundant cellular machinery for viral replication [1]. This was first shown in poliovirus-infected cells [2], and has since been expanded to include human rhinovirus [3], coxsackievirus B3 (CVB3) [4, 5], enterovirus A71 [6], enterovirus D68 [7], and echovirus 7 [8]. Recent findings also highlight an emerging model whereby traditionally non-enveloped enteroviruses, such as poliovirus, CVB3, and enterovirus A71, commandeer the autophagy pathway as conduits for non-lytic viral release [6, 9, 10]. Despite the growing understanding of the enterovirus–autophagy relationship, the interaction between enteroviruses and selective autophagy receptors that govern cargo recruitment to autophagic membranes remains poorly understood.

The autophagy pathway is constitutively active and oscillates between selective and non-selective states to control the quality of proteins/organelles and restore cellular energy. Selective autophagy is mediated by a family of multivalent autophagy receptors that target cytosolic cargo (e.g., misfolded/aggregate proteins, damaged organelles, and invading microbes) to autophagosomes for degradation through the highly conserved ubiquitin association domains that recognize ubiquitinated substrates and the LC3-interaction regions (LIR) that anchor to autophagosomes [11, 12]. Notable autophagy receptors in this family include sequestosome 1 (SQSTM1)/p62, neighbor of BRCA1 (NBR1), optineurin, calcium binding and coiled-coil domain-containing protein 2 (CALCOCO2)/nuclear dot 10 protein 52 (NDP52), and CALCOCO3/Tax1-binding protein 1 (TAX1BP1). All autophagy receptors have been shown to be able to target invading microbes for lysosomal degradation, a process referred to as xenophagy (or virophagy in the context of viral elimination [13]), as part of the cell autonomous innate immune response [14]. However, the role of autophagy receptors in enteroviral infection remains largely unclear. We have previously demonstrated that autophagy receptors SQSTM1 and NBR1 are cleaved following CVB3 infection, leading to impaired clearance of host ubiquitin conjugates [15, 16]; however, the functional significance of this cleavage in CVB3 infection has not been carefully characterized and the role of other autophagy receptors, in particular CALCOCO2/NDP52 that has been extensively studied in bacteria, remains to be investigated in viral propagation.

In the current study, we reported that autophagy receptors SQSTM1 and CALCOCO2 differentially regulate CVB3 replication. We demonstrated that SQSTM1 acts as an antiviral factor, likely by targeting the viral capsid protein for autophagic clearance, whereas CALCOCO2 promotes viral replication through inhibition of the type I interferon signaling. Moreover, we discovered that CVB3 infection induces the cleavage of CALCOCO2, generating the C-terminal cleavage fragment that retains the proviral activity of full-length CALCOCO2.

## Materials and methods

### Cell culture and viral infection

HeLa cells (American Type Culture Collection) were cultured in Dulbecco’s modified Eagle’s medium (DMEM) supplemented with 10% fetal bovine serum (FBS) and a penicillin/streptomycin cocktail (100 µg/ml). CALCOCO2 knockout (KO) HeLa cells were generated through the CRISPR–Cas9 system [17]. The guide RNA sequence targeting exon 6 of CALCOCO2 was 5′-GAAGCAGAACTCAGACATGC-3′, which was cloned into pSpCas9(BB)-2A-Puro vector (Addgene #62988). Following transfection, positive cells were selected using puromycin (7 μg/ml) for up to 2 days. For CVB3 infection, cells were either sham-infected with PBS or inoculated with CVB3 (Kandolf strain) at different multiplicity of infection (MOI) as specified in the figure legends.

### Reagents

The following chemicals were used for the treatment of cells: general caspase inhibitor Z-VAD-FMK (BD Biosciences, #550377), proteasome inhibitor MG132 (Sigma-Aldrich, C2211), lysosome inhibitor bafilomycin A1 (Sigma-Aldrich, B1793), and a synthetic analog of double-stranded RNA, poly-inosinic-cytidylic acid-high molecular weight (poly I:C-HMW Invivogen, #tlrl-pic). The following primary antibodies were used for western blot analysis and/or immunofluorescence staining: CALCOCO2/NDP52 (Santa Cruz Biotechnology, sc-376540), SQSTM1/p62 (PROGEN Biotechnik GmbH, GSQSTM1-C), cleaved caspase-3 (Cell Signaling Technology, #9661), LC3 (MBL International, PM036), VP1 (Dako, M706401-1), NBR1 (Santa Cruz Biotechnology, sc-130380), HA (Roche, 11867423001), Flag (Sigma, F1804), ubiquitin (Cell Signaling Technology, #3933), GFP (Life Technologies, A-6455), K63-ubiquitin (Cell Signaling Technology, #12930), K48-ubiquitin (Cell Signaling Technology, #12805), MAVS (Cell Signaling Technology, #24930), p-TBK1 (Cell Signaling Technology, #5483), TBK1 (Cell Signaling Technology, #3504), LAMP2 (Santa Cruz Biotechnology, sc-8100), ACTB (Sigma-Aldrich, A5316), and GAPDH (Cell Signaling Technology, 14C10).

### Plasmids and small interfering RNA (siRNA)

The myc-tagged wild-type CVB3-3C (3C^wt^) and C147A mutant CVB3-3C (3C^mut^) constructs were generous gifts from Dr. Carolyn Coyne at the University of Pittsburgh [18]. The PHAGE-GFP-CALCOCO2 plasmid was a kind gift from Dr. Richard Youle at the National Institute of Neurological Disorders and Stroke, USA [19]. The 3×Flag-CALCOCO2, 3×Flag-N-CALCOCO2, and 3×Flag-C-CALCOCO2 constructs were generated using CMV10 backbone with the following primers: full-length CALCOCO2 (forward: AAA TTT GAA TTC C ATG GAG GAG ACC ATC AAA GAT C; reverse: AAA TTT GGA TCC TCA GAG AGA GTG GCA GAA CA); N-CALCOCO2 (forward: AAA TTT GAA TTC CAT GGA GGA GAC CAT CAA AG; reverse: AAA TTT GGA TCC TCA CTG AGT GGT AAC AAC C); C-CALCOCO2 (forward: AAA TTT GAA TTC CGG AGA GGT GGA AGA GAT TGA G; reverse: AAA TTT GGA TCC TCA GAG AGA GTG GCA GAA CAC). GFP-CALCOCO2^Q139L^ mutant was generated using the QuikChange Site-Directed Mutagenesis kit (Agilent, #200518) according to the manufacture’s protocol with the following primers: forward: CTG GTT GTT ACC ACT CTG GGA GAG GTG GAA GAG; reverse: CTC TTC CAC CTC TCC CAG AGT GGT AAC AAC CAG. The siRNAs targeting CALCOCO2 (sc-93738) and SQSTM1 (M-010230-00-0005) were purchased from Santa Cruz Biotechnology and Dharmacon, respectively. The scrambled siRNA (sc-37007) was purchased from Santa Cruz Biotechnology. For transfection, HeLa cells were transiently transfected with plasmid cDNAs or siRNAs using Lipofectamine 2000 (Invitrogen, 11668–019) following the manufacturer’s instructions.

### Western blot analysis

Cells were lysed in buffer (10 mM HEPES pH 7.4, 50 mM NaPyrophosphate, 50 mM NaF, 50 mM NaCl, 5 mM EDTA, 5 mM EGTA, 100 µM Na_3_VO_4_, 0.1% Triton X-100) and western blotting was conducted as previously described [20].

### In vitro cleavage assay

In vitro cleavage assay was performed as previously described [21]. Briefly, HeLa cell lysates (30 µg) were incubated with purified wild type or catalytically inactive CVB3 proteinase 3C (0.1 µg) or 2A (0.3 µg) in a cleavage assay buffer (20 mM HEPES pH 7.4, 150 mM potassium acetate, and 1 mM DTT) for 16 h at 37 °C. Reaction was terminated with 6× SDS sample buffer, followed by 95 °C denaturation and subsequent western blot analysis.

### Viral titer measurement

Samples were serially diluted and overlaid on 60-well Terasaki plates of HeLa cells. After 48 h incubation, 50% tissue culture infective dose titer (TCID50) was calculated by the statistical method of Reed and Muench [22]. Titers were expressed as plaque-forming unit (PFU)/ml with one infectious unit equal to 0.7 TCID50 as described previously [23].

### Immunofluorescence and confocal microscopy

After fixation and permeabilization, cells were blocked for 1 h with 3% bovine serum albumin, followed by incubation with primary antibodies at 4 °C overnight and then secondary antibodies for 1 h. After washes, coverslips were mounted using Fluoroshield with DAPI (Sigma-Aldrich, F6057). Images were captured with the Zeiss LSM 880 Inverted Confocal Microscopy.

### Immunoprecipitation

Immunoprecipitation (IP) of VP1 was performed using anti-VP1 antibody (Dako, M706401-1) pre-incubated with Dynabeads Protein G (Invitrogen, 1003D) according to the manufacturer’s instructions. IP of Flag-tagged constructs was performed using EZview^TM^ Red ANTI-FLAG^®^ M2 Affinity Gel (Sigma-Aldrich, F2426) according to the manufacturer’s instructions. In brief, HeLa cell lysates were incubated with anti-Flag M2 agarose beads at 4 °C overnight. After three washes, the bound proteins were eluted with 2× SDS sample buffer and then subjected to western blot analysis.

### Real-time quantitative RT-PCR

Total RNA was extracted using the RNeasy Mini kit (Qiagen, 74104). To determine the expression level of IFN-α and IFN-β, quantitative PCR targeting IFN-α (forward primer: GCC TCG CCC TTT GCT TTA CT; reverse primer: CTG TGG GTC TCA GGG AGA TCA), and IFN-β (forward primer: GTC TCC TCC AAA TTG CTC TC; reverse primer: ACA GGA GCT TCT GAC ACT GA) was performed in a 10-µl reaction containing 1 μg of RNA, using the TaqMan™ RNA-to-CT™ 1-Step Kit (Life Technologies, 4392653) and normalized to GAPDH mRNA according to the manufacturer’s instructions. The PCR reaction was performed on a ViiA 7 Real-Time PCR System (Applied Biosystems). Samples were run in triplicate and analyzed using comparative CT (2^−ΔΔCT^) method with control samples and presented as relative quantitation (RQ).

### Enzyme-linked immunosorbent assay (ELISA)

Human IFN-β in cell culture supernatants  was measured by ELISA through the custom services provided by Eve Technologies (HIFNAB-02-02, Calgary, AB).

### Statistical analysis

Results are presented as mean ± standard deviation (SD) unless otherwise stated. Statistical analysis was performed with unpaired Student’s *t* test. A *P* value <0.05 was considered to be statistically significant. Sample size for all experiments corresponds to three biological replicates. Where statistical significance is evaluated, variance between groups is confirmed to be similar between comparison groups (control vs. experimental) and the statistical analysis is deemed appropriate.

## Results

### CALCOCO2/NDP52 and SQSTM1/p62 differentially regulate CVB3 propagation

**Fig. 1:**
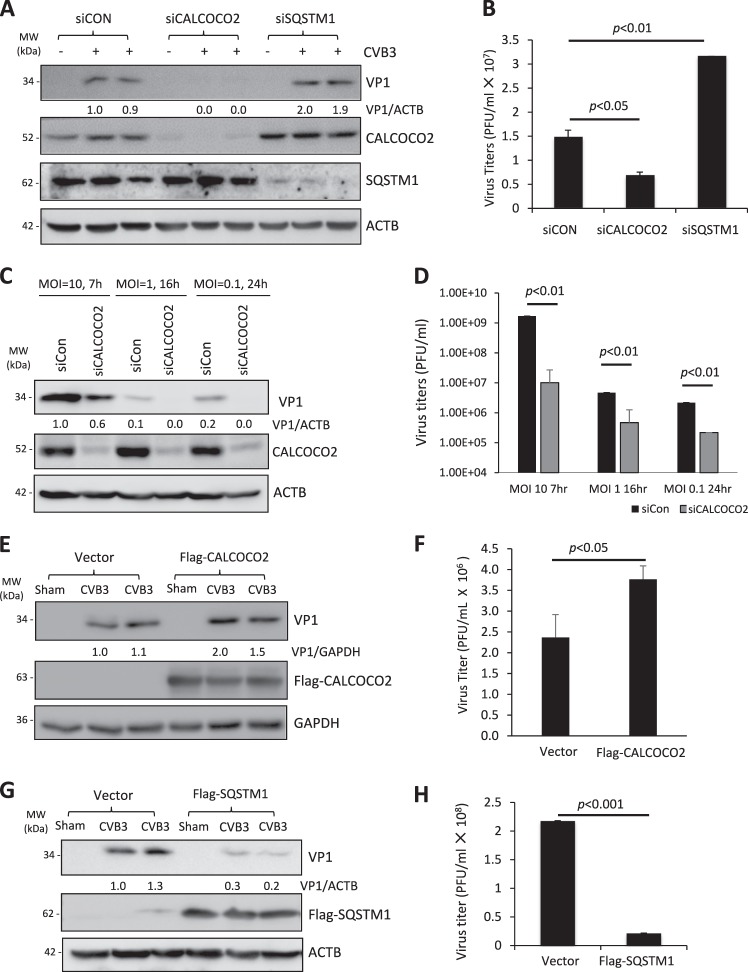
CALCOCO2 and SQSTM1 differentially regulate CVB3 propagation. **a** HeLa cells were transiently transfected with siRNAs targeting CALCOCO2 (siCALCOCO2) or SQSTM1 (siSQSTM1), or a scramble siRNA control (siCON) for 48 h, followed by CVB3 infection (MOI = 0.1) for 24 h. Western blotting was performed to examine protein expression of CALCOCO2, SQSTM1, VP1, and ACTB. Protein levels of VP1 were quantified by densitometric analysis using NIH ImageJ, normalized to ACTB and presented underneath as fold changes compared to sham (the first lane of sham is arbitrarily set a value of 1). **b** HeLa cells were treated as above. Cell-associated virus titers were determined by TCID_50_. Data are represented as mean ± SD from three replicates. **c**, **d** HeLa cells were treated with siCALCOCO2 as above, and then subjected to infection with different doses of CVB3 for various times as indicated. Western blotting and densitometric analysis were conducted (**c**) and virus titers (mean ± SD, *n* = 3) were measured (**d**) as above. **e**–**h** HeLa cells were transfected with constructs overexpressing Flag-CALCOCO2 (**e**, **f**) or Flag-SQSTM1 (**g**, **h**) for 24 h and then subjected to CVB3 infection (MOI = 0.1) for 16 h. Cell lysates were analyzed by western blotting for VP1 protein levels (**e**, **g**) and cell-associated virus titers were quantified as above (**f**, **h**). Results in this figure represent data from two to three independent experiments

To determine the role of autophagy receptors, SQSTM1 and CALCOCO2, in coxsackievirus infection, we first examined the effects of gene silencing of each receptor on viral propagation. As shown in Fig. 1a, b, knockdown of SQSTM1 led to enhanced viral capsid protein (VP1) expression and increased cell-associated viral titers after 24 h infection with a low dose of CVB3 (multiplicities of infection (MOI) of 0.1). In contrast, depletion of CALCOCO2 caused a significant attenuation of viral growth. Given the possible impacts of different viral dosages and infectious cycles, we further confirmed our observations in CALCOCO2-depleted cells by treating the cells with three logarithmic doses of CVB3 (MOI of 10, 1, and 0.1) for three respective time points (7, 16, and 24 h) that spanned three viral replication cycles. In agreement, results from all three conditions concluded that depletion of CALCOCO2 resulted in a concomitant decrease of viral protein synthesis as well as infectious viral titers (Fig. 1c, d). In line with these observations, ectopic expression of 3×Flag-tagged CALCOCO2 showed an enhancement in both VP1 expression and intracellular viral titers (Fig. 1e, f), whereas cells expressing exogenous Flag-SQSTM1 exhibited a reduction in both metrics (Fig. 1g, h). Collectively, our results demonstrated opposing roles for SQSTM1 and CALCOCO2 in CVB3 infection.

### Both SQSTM1 and CALCOCO2 physically interact with CVB3 capsid protein VP1

**Fig. 2:**
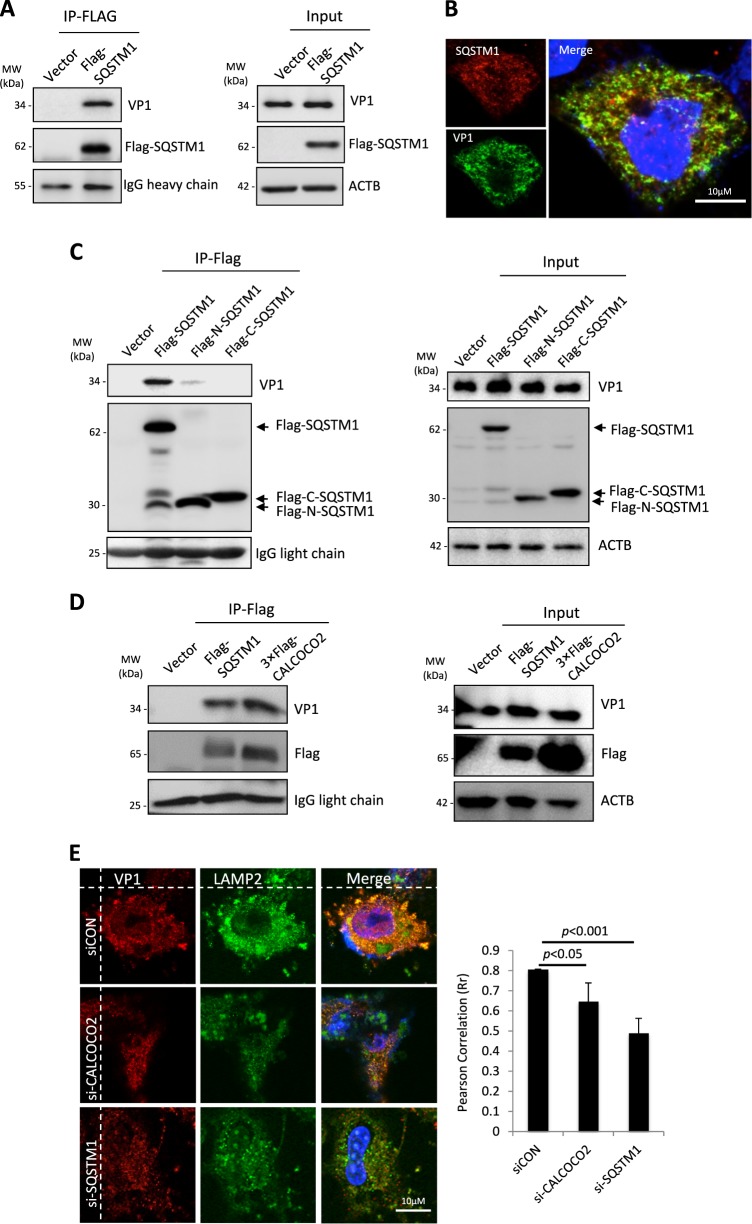
SQSTM1 and CALCOCO2 physically interact with CVB3 capsid protein VP1. **a** HeLa cells were transfected with Flag-tagged SQSTM1 for 24 h, followed by CVB3 infection (MOI = 10) for 5 h. After immunoprecipitation (IP) with an anti-Flag antibody, western blotting was performed for detection of viral capsid protein 1 (VP1) and Flag-SQSTM1. Blots for antibody IgG heavy chain and ACTB were shown as loading controls for IP and input, respectively. **b** Confocal microscopy analysis of the co-localization of endogenous SQSTM1 and viral capsid protein VP1. HeLa cells were infected with CVB3 (MOI = 10) for 3 h. Cells were then fixed and immunostained for SQSTM1 and VP1. The nucleus was stained with DAPI. **c**, **d** HeLa cells were transfected with Flag-SQSTM1, Flag-N-SQSTM1, Flag-C-SQSTM1, 3×Flag-tagged CALCOCO2, or empty vector as indicated for 24 h, followed by CVB3 infection (MOI = 10) for 5 h. Co-immunoprecipitation (*Co-IP) and western blotting were conducted as above in **a**. Blots for IgG light chain and ACTB were used as loading controls for IP and input, respectively. Similar results were observed in two independent experiments. **e** Confocal microscopy analysis of the co-localization between VP1 and LAMP2. HeLa cells were treated with control siRNA (siCON), siCALCOCO2, or siSQSTM1 for 48 h, followed by CVB3 infection (MOI = 10) for 3 h. Cells were then immunostained for VP1 and LAMP2. The nucleus was stained with DAPI. Co-localization between VP1 and LAMP2 was quantified using ImageJ and presented as Pearson correlation (Rr)

Previous studies have shown that SQSTM1 can interact with capsid proteins of Sindbis virus [24] and Chikungunya virus [25]. We therefore questioned whether CVB3 capsid proteins are also recognized by SQSTM1. HeLa cells transiently expressing Flag-SQSTM1 were infected with CVB3 at an MOI of 10 for 5 h, followed by co-immunoprecipitation using anti-Flag M2 beads. Figure 2a showed that Flag-SQSTM1, but not a vector control, was able to immunoprecipitate viral capsid protein, VP1. Likewise, immunostaining revealed the co-localization of viral capsid protein and SQSTM1 in CVB3-infected cells (Fig. 2b). We have previously demonstrated that SQSTM1 is cleaved before glycine 241 during CVB3 infection through the activity of CVB3-encoded proteinase 2A [16]. Here we found that the binding affinity to VP1 for both the resulting N- and C-terminal cleavage fragments of SQSTM1 was markedly decreased (Fig. 2c), indicating that cleavage of SQSTM1 may serve as a viral strategy to counteract the role of SQSTM1 in viral clearance. Furthermore, immunoprecipitation also revealed an interaction between CALCOCO2 and viral protein VP1 (Fig. 2d). Finally, confocal co-localization studies demonstrated that depletion of SQSTM1 and CALCOCO2 in virus-infected cells impaired the association of VP1 with the lysosome and late endosome marker, LAMP2 (Fig. 2e). Together, our data suggest a potential role for SQSTM1 and CALCOCO2 in virophagy through interacting with viral capsid proteins and by facilitating recruitment of VP1 to lysosomes/late endosomes.

### CVB3 capsid protein VP1 undergoes ubiquitination

**Fig. 3:**
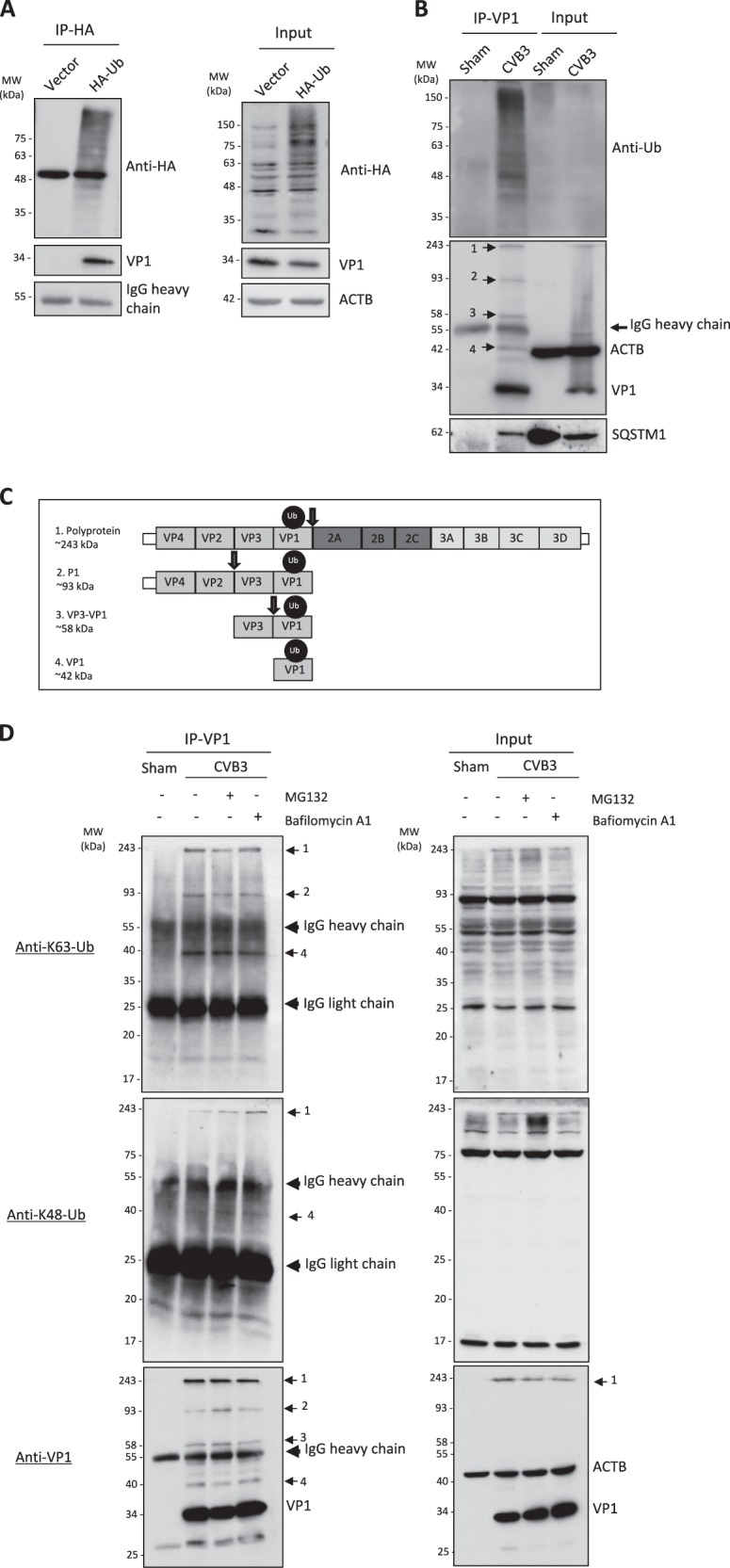
CVB3 capsid protein VP1 undergoes ubiquitination. **a** HeLa cells were transiently transfected with HA-tagged ubiquitin (HA-Ub) or empty vector for 24 h, and then infected with CVB3 (MOI = 10) for 5 h. Co-IP was performed with an anti-HA antibody, followed by western blot analysis of HA-Ub using antibodies against HA-tag or VP1. **b** HeLa cells were infected with CVB3 (MOI = 10) for 5 h, followed by IP using anti-VP1 antibody. Western blotting was conducted using antibodies against Ub, VP1, or SQSTM1 as indicated. **c** Schematic illustration of the CVB3 polyprotein and the respective VP1-linked ubiquitinated fragments. Arrows depict sites of cleavage by virus-encoded proteinases. **d** HeLa cells were infected with CVB3 (MOI = 10) for 5 h in the presence or absence of a proteasome inhibitor MG132 (10 μM) or a lysosome inhibitor bafilomycin A1 (200 nM) as indicated. IP was conducted using an anti-VP1 antibody, followed by western blotting was performed using anti-K63-linked or anti-K48-linked Ub antibody and anti-VP1 antibody as indicated. Similar results were observed in three independent experiments

Since autophagy receptors recognize the targets via their ubiquitin association domains, we then questioned whether ubiquitin or ubiquitination process plays a role in linking SQSTM1/CALCOCO2 with viral capsid protein. To test this model, we initially examined whether VP1 itself can associate with ubiquitin by performing co-immunoprecipitation of exogenously expressed HA-ubiquitin in CVB3-infected HeLa cells. We found that VP1 indeed interacted either directly or indirectly, with ubiquitin or ubiquitinated proteins (Fig. 3a). Moreover, reverse immunoprecipitation of VP1 from CVB3-infected HeLa cells revealed a significant pulldown of ubiquitinated species, which may include ubiquitinated VP1 and other viral and host ubiquitinated proteins (Fig. 3b). Of note, immunoprecipitation of VP1 routinely showed the presence of four distinct protein bands in immunoblots incubated with anti-VP1 antibody (Fig. 3b). We concurred that these four distinct bands likely represent VP1-positive fragments, which are generated following viral processing of the polyprotein (Fig. 3c). To further characterize the status of ubiquitinated species observed following VP1 immunoprecipitation, we utilized the ubiquitin-specific antibodies, which recognize lysine-63 (K63) or lysine-48 (K48) linkages, in the presence or absence of the lysosome (bafilomycin A1) or proteosome (MG132) inhibitors. Western blotting with anti-K63-ubiquitin or anti-K48-ubiquitin antibody showed the detection of distinct protein bands that were unresponsive to degradative blockade and migrated at the same molecular weight as those observed following VP1 immunoprecipitation, suggesting that the ubiquitin-specific antibodies recognize VP1-associated fragments (Fig. 3d). Our data indicate a possible mechanism by which autophagy receptors recognize the invading viral pathogens through association with ubiquitinated viral capsid proteins.

### CALCOCO2 attenuates type I interferon signaling

**Fig. 4:**
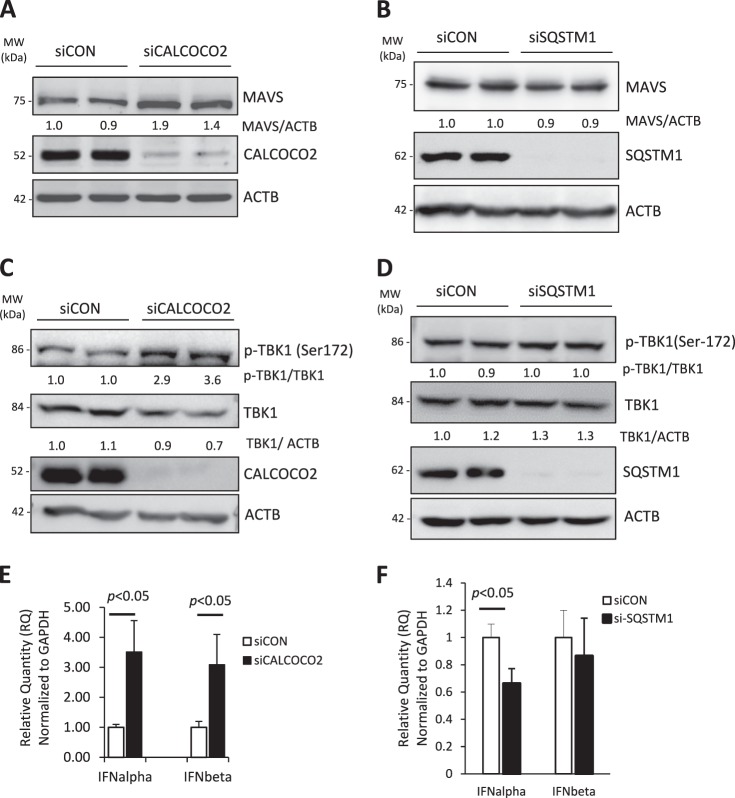
Knockdown of CALCOCO2 results in increased protein levels of MAVS and p-TBK1 as well as elevated production of type I interferons. **a**, **c**, **e** HeLa cells were treated with siCALCOCO2 or siCON for 48 h. Cell lysates were harvested and probed for MAVS (**a**) and p-TBK1/TBK1 (**c**) by western blotting. Knockdown efficiency was verified by probing with anti-CALCOCO2 antibody. Densitometry was carried out as in Fig. 1. Total RNA was extracted and real-time qRT-PCR was performed to measure mRNA levels of IFN-α and IFN-β normalized to GAPDH mRNA (**e**), and presented as relative quantification (RQ) with respect to siCON (RQ ± SE, *n* = 3, confidence interval at 95%). **b**, **d**, **f** HeLa cells were transfected with siSQSTM1 or siCON for 48 h, followed by western blot analysis of MAVS (**b**) and p-TBK1/TBK1 (**d**) and qRT-PCR measurement of IFN-α and IFN-β (**f**) as above. Similar results were observed in three independent experiments

Despite the possible role of CALCOCO2 in virophagy, the overall function of CALCOCO2 is to enhance CVB3 infection (Fig. 1). Thus, we next sought to determine the proviral mechanism of CALCOCO2. Type I interferon (IFN) signaling is a broad antiviral pathway and was previously reported to inhibit CVB3 replication [26, 27]. Recent studies have suggested an important role for CALCOCO2 in the deactivation of type I IFN signaling through autophagy-mediated clearance of the mitochondrial antiviral signaling (MAVS) [28]. To test the hypothesis that CALCOCO2 facilitates CVB3 replication by inhibiting the type I IFN response, we examined the effects of knockdown of CALCOCO2 on the protein levels of MAVS, phosphorylation of TANK-binding kinase 1 (TBK1, a downstream target of MAVS [29]), and mRNA production of IFN-α/IFN-β. We showed that depletion of CALCOCO2 led to increased protein accumulation of MAVS (Fig. 4a) and elevated phosphorylation of TBK1 (Fig. 4c). No evident effects of knockdown of SQSTM1 on MAVS accumulation (Fig. 4b) and TBK1 phosphorylation (Fig. 4d) were observed. Lastly, quantitative PCR results demonstrated that gene silencing of CALCOCO2, but not SQSTM1, caused increased mRNA levels of IFN-α/IFN-β (Fig. 4e, f). Taken together, our data suggest that CALCOCO2 acts as a suppressor of type I IFN signaling, which likely contributes to its proviral activity.

### CALCOCO2 is cleaved after Q139 following CVB3 infection by viral proteinase 3C

**Fig. 5:**
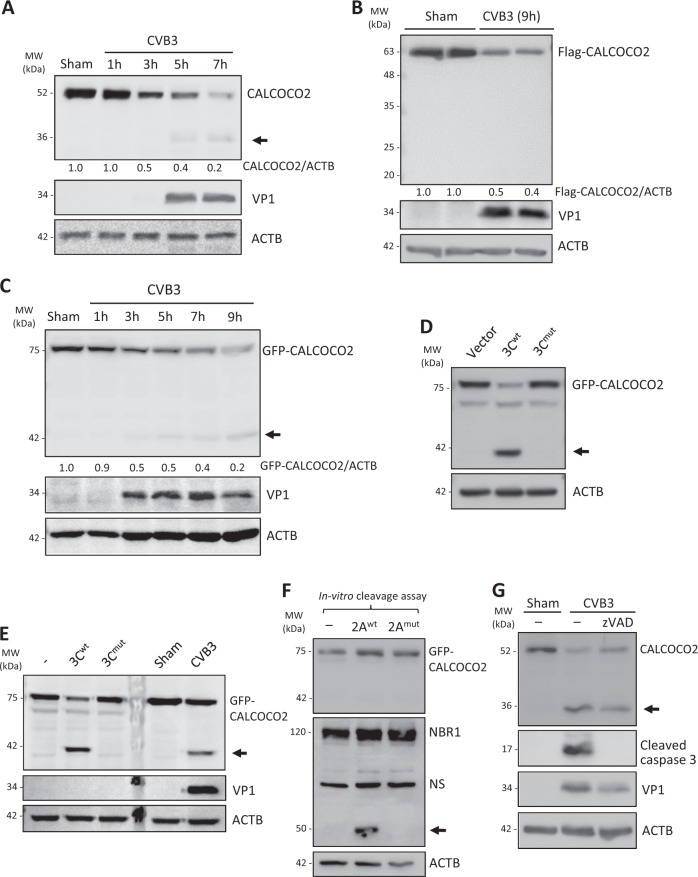
CALCOCO2 is cleaved following CVB3 infection by viral proteinase 3C. **a**–**c** HeLa cells (**a**), HeLa cells transfected with 3×Flag-CALCOCO2 (**b**), or GFP-CALCOCO2 (**c**) for 24 h, were sham- or CVB3-infected (MOI = 10) for the indicated time points. Western blotting was conducted for detection of CALCOCO2 using anti-CALCOCO2 that recognizes the C-terminal region of CALCOCO2 (**a**), anti-Flag (**b**), or anti-GFP antibody (**c**). Densitometry was carried out as in Fig. 1. **d** HeLa cells were transfected with GFP-CALCOCO2, together with either empty vector, wild-type 3C (3C^wt^), or catalytically inactive 3C (C147A) mutant (3C^mut^). After 24 h, cells lysates were collected and analyzed by western blotting with an anti-GFP antibody. **e** In vitro cleavage assay was performed by incubation of lysates (30 μg) from HeLa cells transfected with GFP-CALCOCO2 with vehicle (−), purified 3C^wt^, or 3C^mut^ proteins (0.1 μg) for 16 h. Sham- and CVB3-infected (MOI = 10, 7 h) HeLa cell lysates were included (right two lanes) as a control. Cleavage products of CALCOCO2 were analyzed by western blotting with anti-GFP antibody. **f** In vitro cleavage assay was conducted as above with vehicle (−), recombinant 2A^wt^, or 2A^mut^ proteins (0.3 μg) for 16 h. Cleavage of NBR1 by 2A, which was previously demonstrated [15], was shown as evidence of the activity of 2A. **g** HeLa cells were infected with CVB3 (MOI = 10) for 7 h in the presence or absence of a pan-caspase inhibitor z-VAD-FMK (zVAD, 50 µM) or vehicle (−). Western blotting was then performed with an anti-CALCOCO2 antibody. Activation of caspase-3 was examined using an anti-cleaved caspase-3 antibody. Results in this figure represent data from three independent experiments

**Fig. 6:**
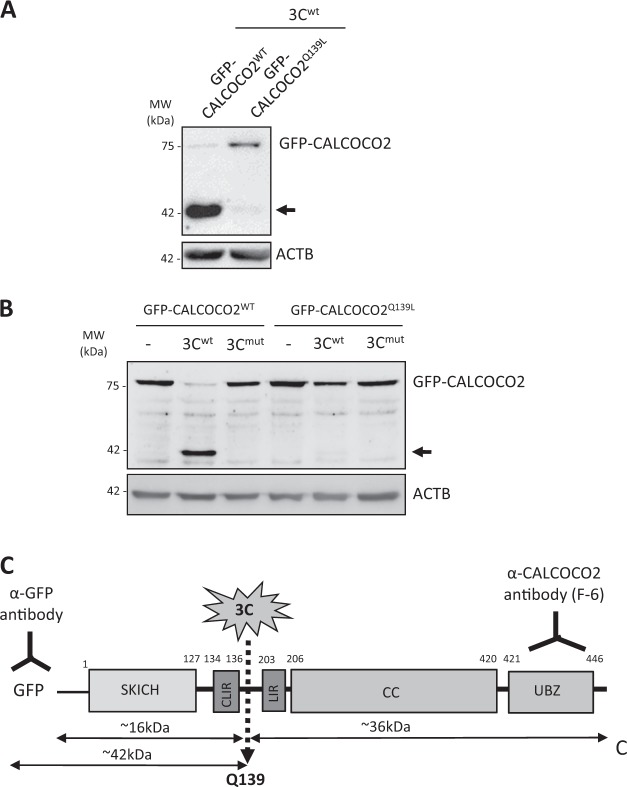
CALCOCO2 is cleaved after Q139 by viral proteinase 3C. **a** HeLa cells were transfected with 3C^wt^ together with GFP-CALCOCO2^WT^ or GFP-CALCOCO2^Q139L^ as indicated. After 24 h, cell lysates were harvested and subjected to western blot analysis using an anti-GFP antibody. **b** In vitro cleavage assay was conducted by incubation of lysates of HeLa cells transfected with GFP-CALCOCO2^WT^ or GFP-CALCOCO2^Q139L^ with recombinant 3C^wt^, 3C^mut^, or vehicle (−) as above in **e**. **c** Schematic illustration of the structural domains, the identified cleavage site, the antibody recognition regions, and the resulting cleavage products of CALCOCO2. SKICH skeletal muscle and kidney-enriched inositol phosphatase (SKIP) carboxyl homology, CLIR LC3C-interacting region, LIR LC3-interacting region, CC coiled-coil domain, UBZ ubiquitin-binding zinc finger region. Arrows denote the cleavage fragments. NS non-specific bands. Results in this figure represent data from two independent experiments

During our characterization of CALCOCO2 as a proviral autophagy receptor, we made the surprising observation that CVB3 infection resulted in a marked reduction in CALCOCO2 protein levels, accompanied by the appearance of a ~36-kDa protein fragment using an anti-CALCOCO2 antibody targeting to the C-terminal region (Fig. 5a). This result suggests a likely cleavage of CALCOCO2. In line with this, HeLa cells transiently expressing 3×Flag-CALCOCO2 showed an equally significant decrease in full-length CALCOCO2 levels following CVB3 infection, although no apparent fragment was observed with the N-terminal anti-Flag antibody (Fig. 5b). Further ectopic expression using an N-terminally tagged GFP-CALCOCO2 demonstrated a decrease in full-length GFP-CALCOCO2 and the detection of an additional band at ~42 kDa using anti-GFP antibody (Fig. 5c), corresponding to an N-terminal cleavage fragment of CALCOCO2 which complemented our previously observed endogenous CALCOCO2 C-terminal fragment (Fig. 6c).

We then studied whether CVB3-encoded proteinases are responsible for the cleavage of CALCOCO2. Co-transfection of GFP-CALCOCO2 with constructs expressing viral proteinase 3C^wt^, but not a catalytically inactive 3C mutant (3C^mut^), led to the production of the same ~42 kDa N-terminal cleavage fragment, as that observed following CVB3 infection (Fig. 5d). Similarly, in vitro cleavage assays using recombinant proteinases 3C^wt^, but not 3C^mut^, showed the detection of CALCOCO2 cleavage fragment (Fig. 5e), suggesting that viral proteinase 3C is responsible for the cleavage of CALCOCO2. To rule out the possible involvement of viral proteinase 2A and host caspases, which are activated during CVB3 infection, we conducted an in vitro cleavage assay using recombinant 2A^wt^ or 2A^mut^ (Fig. 5f) and western blotting using the pan-caspase inhibitor zVAD-fmk (Fig. 5g). We found that addition of viral proteinase 2A^wt^ or inhibition of general caspase activity failed to induce the cleavage of CALCOCO2, indicating that CALCOCO2 cleavage is independent of 2A and caspase activation.

Having identified that CALCOCO2 is cleaved by viral proteinase 3C, we performed site-directed mutagenesis based on the enteroviral 3C consensus sequence [30] to identify the potential cleavage sites on CALCOCO2. Both in vivo (in cells transfected with 3C^wt^ proteinase construct, Fig. 6a) and in vitro (using recombinant purified viral proteinase, Fig. 6b) cleavage assay showed that GFP-CALCOCO2^Q139L^ (glutamine 139 mutated to leucine) is resistant to 3C-mediated cleavage, suggesting that CALCOCO2 is cleaved by viral proteinase 3C after Q139. This cleavage separates the N-terminal skeletal and kidney-enriched inosital phosphatase (SKIP) carboxyl homology (SKICH) and LC3C-interacting region (CLIR) of CALCOCO2 from its C-terminal LIR, coiled-coil (CC), and ubiquitin-binding zinc finger (UBZ) domains (Fig. 6c).

### The C-terminal cleavage fragment of CALCOCO2 retains the function of full-length CALCOCO2 in promoting viral replication

**Fig. 7:**
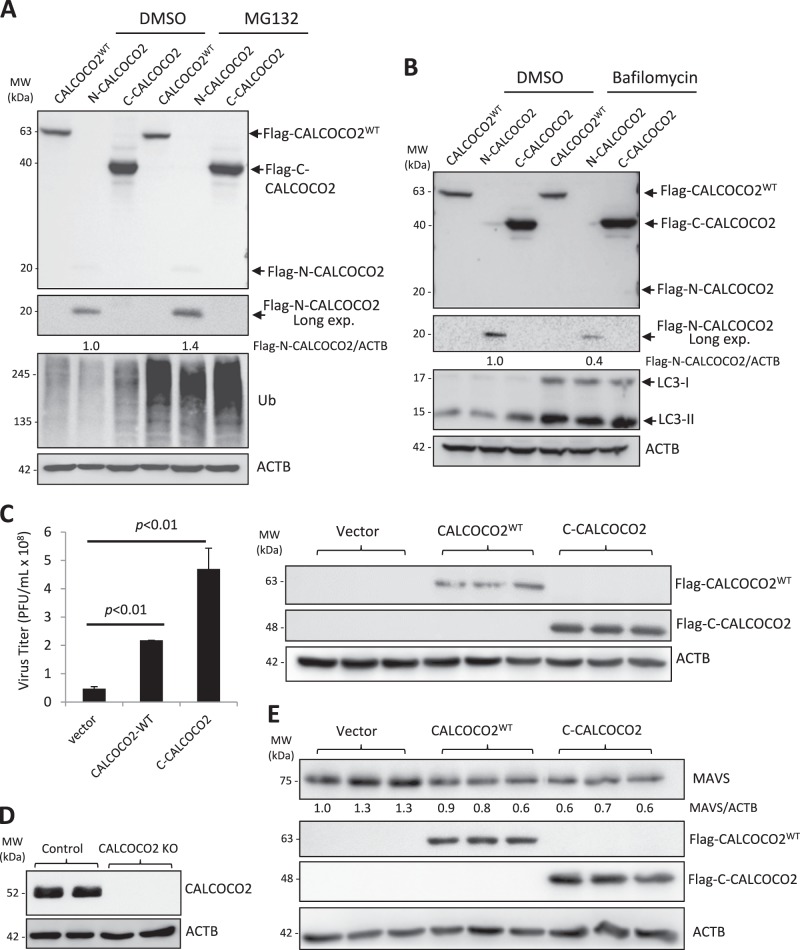

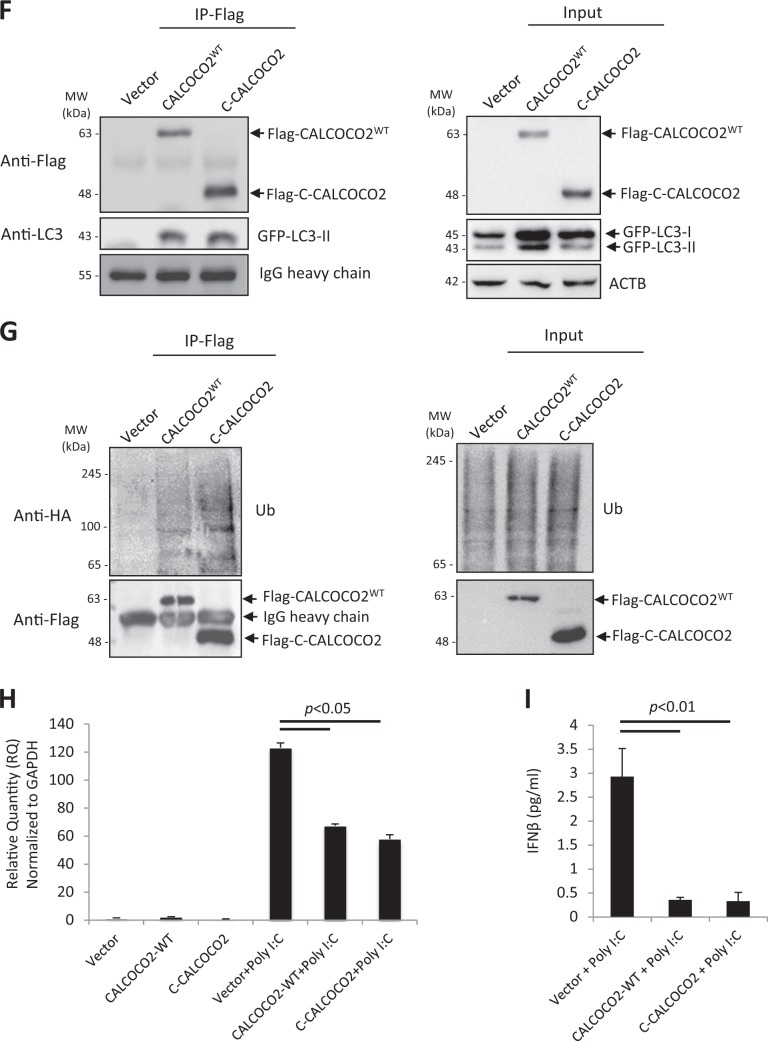
The C-terminal cleavage fragment of CALCOCO2 retains the function of full-length CALCOCO2 in promoting CVB3 growth. **a**, **b** HeLa cells were transfected with 3×FLAG-tagged CALCOCO2^WT^, N- or C-terminal cleavage fragments of CALCOCO2 (N-CALCOCO2 or C-CALCOCO2) in the presence of vehicle (DMSO), proteosomal inhibitor (MG132, 10 µM) (**a**), or lysosome inhibitor (bafilomycin, 200 nM) (**b**) for 6 h. Protein levels of full length and the respective cleavage fragments of CALCOCO2 were verified by western blotting with an anti-Flag antibody. Ubiquitin (**a**) and LC3 (**b**) were probed by western blotting to confirm the inhibition of proteasome and lysosome activities, respectively. Densitometry was conducted as in Fig. 1. **c** HeLa cells were transfected with 3×FLAG-tagged CALCOCO2^WT^, C-CALCOCO2, or empty vector for 24 h, followed by CVB3 infection (MOI = 10) for 7 h. Virus titer (mean ± SD, *n* = 3) were measured by TCID_50_ and western blotting was performed to confirm the expression of exogenous CALCOCO2 using an anti-Flag antibody. **d** Knockout (KO) efficiency of the CALCOCO2 KO HeLa cells was verified by western blotting with anti-CALCOCO2 antibody. **e** CALCOCO2 KO cells were transfected with empty vector, 3×FLAG-tagged CALCOCO2^WT^ or C-CALCOCO2 for 16 h. Western blot analysis was conducted to examine the protein expression of MAVS, Flag-CALCOCO2, and ACTB. Protein levels of MAVS were quantitated by densitometric analysis, normalized to ACTB, and presented as fold changes (mean ± SD, *n* = 3) compared with vector control. **f**, **g** HeLa cells were co-transfected with empty vector, 3×FLAG-tagged CALCOCO2^WT^ or C-CALCOCO2, together with either GFP-LC3 (**f**) or HA-Ubiquitin (**g**) for 16 h. Co-IP and western blotting were conducted as above. Blots for IgG heavy chain and ACTB were used as loading controls for IP and input, respectively. Results represent data from two independent experiments. **h**, **i** CALCOCO2 KO HeLa cells were transfected with empty vector, 3×FLAG-tagged CALCOCO2^WT^ or C-CALCOCO2 for 16 h, followed by addition of poly I:C (1 μg/ml) for another 12 h. Cells were collected for RNA extraction and qRT-PCR was conducted to determine the mRNA levels of IFN-β (H, mean ± SD, *n* = 3). Culture supernatants were harvested for the measurement of IFN-β secretion by ELISA (I, mean ± SD, *n* = 3)

We next sought to evaluate the functional consequence of CALCOCO2 cleavage by generating recombinant fragments corresponding to the N-terminal (N1-139) and C-terminal (C140-446) cleavage products. Of note, we found that the N-CALCOCO2 fragment was less stable as compared to full-length and C-CALCOCO2 fragment (Fig. 7a, b), consistent with the finding that the Flag-N-CALCOCO2 was undetectable following CVB3 infection (Fig. 5b). Further experiments using proteasome and lysosome inhibitors showed that the N-CALCOCO2 fragment was degraded mainly through the proteasome pathway, as evidenced by the accumulation of N-CALCOCO2 in the presence of proteasome inhibitor MG132 (Fig. 7a), but not lysosome inhibitor bafilomycin A1 (Fig. 7b). Based on these findings, our study hereafter focused on the C-CALCOCO2 fragment to further elucidate its functional significance. Similar to full-length CALCOCO2^WT^, we found that expression of C-CALCOCO2 caused a significant increase in viral titers (Fig. 7c), suggesting that viral cleavage of CALCOCO2 does not impair its proviral activity.

We then further determined the proviral mechanism of C-CALCOCO2. To rule out the possible interference from endogenous CALCOCO2, we generated CALCOCO2 knockout cells using the CRISPR–Cas9 gene editing system (Fig. 7d). The cells were then reconstituted with a vector control, CALCOCO2^WT^, or C-CALCOCO2 by transient transfection. As shown in Fig. 7e, expression of C-CALCOCO2, similar to CALCOCO2^WT^, led to a marked decrease in protein levels of MAVS, indicating that the C-terminal cleavage fragment is still able to degrade MAVS. We further examined whether C-CALCOCO2, which lost the N-terminal SKICH (skeletal muscle and kidney-enriched inositol phosphatase (SKIP) carboxyl homology) and CLIR (LC3C-interacting region) domains of CALCOCO2^WT^ (Fig. 6c), retains its capacity to act as an autophagy receptor by evaluating its ability to interact with LC3 and ubiquitin. Figure 7f, g demonstrated that both WT- and C-CALCOCO2 were able to interact with exogenously expressed GFP-LC3 and HA-ubiquitin. Finally, we assessed the effects of C-CALCOCO2 on type I IFN production. Since basal level of type I IFNs is low in HeLa cells, the cells were treated with poly I:C, a synthetic analog of double-stranded RNA, to induce the production of type I IFNs (Fig. 7h, i). We found that expression of CALCOCO2^WT^ or C-CALCOCO2 led to a significant decrease in both mRNA levels (Fig. 7h) and protein secretion of IFN-β (Fig. 7i). Collectively, our results support a proviral mechanism of the C-CALCOCO2 fragment through attenuating the type I IFN signaling by targeting the MAVS protein.

## Discussion

Enterovirus replication is tightly associated with host cellular machinery, including the autophagy pathway, which has been previously shown to be beneficial for viral growth by providing physical scaffolds for viral replication [2, 4, 5], and through facilitating autophagosome-associated non-lytic viral spread [6, 9, 10]. Additional support was provided from a recent study, which used a high-throughput genetic screen to identify autophagy and membrane trafficking genes as critical regulators of susceptibility to *Picornaviruses* infection [31]. We have previously demonstrated that CVB3 infection promotes the formation of autophagosomes while at the same time targeting several autophagy receptor and adapter proteins, including SQSTM1/p62, NBR1, synaptosomal-associated protein 29 (SNAP29), and pleckstrin homology domain-containing family M member 1(PLEKHM1) to disrupt its degradative capacity [5, 15, 16, 20]. The current study provides further insights into the mechanism by which CVB3 subverts autophagy by identifying the autophagy receptor CALCOCO2 as a novel viral substrate that is co-opted to facilitate viral propagation.

CALCOCO2 is generally considered a xenophagic receptor; this is mainly based on previous evidence on bacteria, most notably Salmonella [32–34]. For viral infection, it was previously reported that CALCOCO2 binds the non-structural protein 2 of Chikungunya virus to promote viral replication in humans but not mice, suggesting not only a proviral function but also a species-specific role for CALCOCO2 [25]. Other studies also showed a proviral role for CALCOCO2 in Influenza and Measles viral infection [35, 36]. However, the mechanism has not been investigated and CALCOCO2 interaction with various viral proteins has been proposed to be relevant [25, 35, 36]. In this study, our results support that in addition to its function in selective autophagy, CALCOCO2 plays a dual role in CVB3 infection through facilitating virophagy and by impairing the host antiviral response; but the overall activity of CALCOCO2 is to enhance viral replication by compromising the type I IFN response. Type I IFN signaling provides a potent host innate immunity against enteroviral infection. Indeed, CVB3 has been shown to disrupt this pathway through virus-mediated cleavage of MAVS protein [37, 38]. Consistent with a recent report [28], we found that loss of CALCOCO2 causes increased accumulation of MAVS and phosphorylation of TBK1, a prerequisite for the downstream production of IFN α/β cytokines. We also showed that depletion of CALCOCO2, but not SQSTM1, results in an elevated production of IFN-α/IFN-β mRNA, providing a plausible mechanism by which autophagy receptors differentially regulate CVB3 replication.

CALCOCO2 is cleaved during CVB3 infection by viral proteinase 3C, generating the stable form of the C-terminal cleavage fragment. Despite the loss of the N-terminal CLIR domain, the C-CALCOCO2 maintains its ability to interact with LC3, likely through the remaining LIR (Fig. 6c). We further demonstrated that the C-CALCOCO2 fragment is still able to target MAVS and attenuates the type I IFN signaling.

We previously reported that perturbation of SQSTM1, either through genetic silencing or exogenous overexpression, does not contribute to viral replication [16]. The current study addresses the limitations of our previous research, in which viral propagation was assessed after challenging cells with a high dose of CVB3, which we now realize may fail to capture the subtle nuances of viral replication. The present study utilized a low dose of CVB3 infection to re-examine the role of SQSTM1 in viral propagation and demonstrated an antiviral function of SQSTM1.

It was first identified by Beth Levine’s laboratory that SQSTM1 binds and targets the capsid proteins of Sindbis virus for autophagic degradation [24]. Later, SQSTM1 interaction with capsid proteins was also reported during Chikungunya viral infection [25]. Our current study revealed that both SQSTM1 and CALCOCO2 are able to interact with CVB3 capsid protein VP1. We further demonstrated that VP1 undergoes ubiquitination, a substrate signal recognized by autophagy receptors, but whether intact infectious capsids are also ubiquitinated remains to be determined. Autophagy receptors can initiate virophagy either in a ubiquitin-dependent or independent manner [39]. With regard to SQSTM1, both ubiquitin-dependent and independent targeting have been reported for Chikungunya and Sindbis virus [24, 25]. An important consideration in virophagy is the nature of ubiquitin association, i.e., whether autophagy substrates, such as viral capsid proteins, are either directly ubiquitinated via the activity of E3 ubiquitin ligases, or rather indirectly associate with host ubiquitinated factors. In both cases, the presence of ubiquitin can signal for the recruitment of autophagy receptors [40]. But an additional consideration should be made for the former in which direct ubiquitin conjugation can occur through various linkages. Using linkage-specific ubiquitin antibodies, our study revealed that CVB3 capsid proteins can exhibit multiple forms, including K48 and/or K63-linked ubiquitination by a yet unidentified E3 ligase. One potential candidate, the E3 ligase SMURF1 (Smad ubiquitin regulatory factor 1), was previously shown to not only interact with SQSTM1, but to also regulate virophagy of Sindbis and herpes simplex virus, albeit in a ubiquitin conjugation-independent manner [41]. Another likely candidate is the SQSTM1-associated E3 ligase TRAF6 (tumor necrosis factor receptor (TNF)-associated factor 6), which plays an important role in antiviral signaling via the NF-κB pathway. Of note, our previous report demonstrated that CVB3-mediated cleavage of SQSTM1 occurs within its respective TRAF6-binding domain, leading to disrupted NF-κB signaling [16]. In addition to CVB3, cleavage of SQSTM1 has recently been confirmed upon poliovirus, rhinovirus, and enterovirus D68 infection [7], suggesting a common enteroviral strategy to counteract selective autophagy receptor.

In summary, our study provides novel insight into the mechanisms by which CVB3 evades host virophagy, through viral-mediated cleavage of CALCOCO2 and SQSTM1, to promote viral propagation.
